# The association of coital incontinence with clinical factors and single voiding cycle ambulatory urodynamic monitoring findings in women

**DOI:** 10.3389/fmed.2023.1160637

**Published:** 2023-03-28

**Authors:** Şerife Esra Çetinkaya, Mehmet Murat Seval, Bulut Varlı, Fulya Dökmeci

**Affiliations:** Department of Obstetrics and Gynecology, Faculty of Medicine, Ankara University, Ankara, Turkey

**Keywords:** coital incontinence, clinical risk factors, ambulatory urodynamic monitoring, stress urinary incontinence, detrusor overactivity

## Abstract

**Introduction:**

Coital incontinence (CI) is a frequent problem in women with urinary incontinence (UI) with significant impact on female sexuality and quality of life. The underlying mechanism is controversial; it has been known that CI is associated with both stress urinary incontinence (SUI) and detrusor overactivity (DO). However, recently it has been reported that CI is mainly related with SUI and urethral incompetence, but not with DO. Ambulatory urodynamic monitoring (AUM) has been shown to be a sensitive tool for the detection of DO. The aim of this study was to investigate the clinical risk factors for CI and the association of CI with urodynamic diagnoses at single voiding cycle AUM.

**Methods:**

Records of sexually active women with urinary incontinence attending the urogynaecology unit of a university hospital, who completed the PISQ-12 were reviewed retrospectively (*n* = 1,005). Patients were grouped using the 6th question; patients answering “never” to this question were considered as continent during coitus (*n* = 591) and patients reporting any urinary leakage at coitus were considered to have CI (*n* = 414). Demographics, clinical examination findings, incontinence severity measured by the Sandvik Incontinence Severity Index, scores of Turkish validated questionnaires (PFDI-20, IIQ-7, OAB-V8, and PISQ-12) and single voiding cycle AUM findings were compared, and univariate and multivariate logistic regression analyses were performed.

**Results:**

Among all sexually active women with UI, 41.2% had CI; UI was more severe, symptom bother was higher, related quality of life (*p* < 0.001) and sexual function were worse (≤0.018) in these women. Younger age (OR 0.967, *p* < 0.001), history of vaginal delivery (OR 2.127, *p* = 0.019), smoking (OR 1.490, *p* = 0.041), postural UI (OR 2.012, *p* = 0.001), positive cough stress test (OR 2.193, *p* < 0.001), and positive SEST (OR 1.756, *p* = 0.01) were found as independent clinical factors associated with CI. Urodynamic SUI (OR 2.168, *p* = 0.001) and MUI (OR 1.874, *p* = 0.002) were found as significant and independent urodynamic diagnoses associated with CI, whereas no association was found with DO or UUI.

**Conclusion:**

Both clinical and AUM findings supported that CI is a more severe form of UI that it is mainly related with SUI and urethral incompetence, but not with UUI or DO.

## Introduction

Coital incontinence (CI), which is defined as involuntary loss of urine at coitus, has been reported to have significant impact on female sexuality and quality of life (1–3). Although women rarely state it as their presenting complaint, it is a frequent problem in the urogynecologic clinical setting upon direct questioning and with the use of validated questionnaires; a wide range of prevalence between 10 to 66% have been reported among sexually active women with urinary incontinence (UI) (3–8).

Conflicting results exist in the literature regarding the risk factors of CI and its underlying pathophysiologic mechanism (4, 5, 8–10). Limited studies have investigated the relation of CI with urodynamic diagnoses; some studies have reported that CI at penetration is mainly associated with stress urinary incontinence (SUI), and that CI at orgasm is mainly associated with detrusor overactivity (DO) (6, 7). However, recently it has been reported that CI is mainly related with SUI and urethral incompetence, but not with DO (5, 8). On the other hand, it has also been claimed that there is no relation between urodynamic diagnoses and CI during neither penetration nor orgasm (3).

Ambulatory urodynamic monitoring (AUM) is considered a valuable second line diagnostic tool enabling evaluation and discrimination of complicated lower urinary tract symptoms (LUTS) in a physiological setting; it has been shown to be more sensitive in the detection of DO, providing additional information that may change management, when symptoms cannot be reproduced at conventional urodynamics (11–15). Indeed, it has been demonstrated to detect DO in 50 to 60% of women with overactive bladder (OAB) symptoms when conventional urodynamics was inconclusive (13, 14). It is mainly criticized as a complex and time-consuming procedure lasting for about 3–4 voiding cycles; however, AUM is performed in our department at single voiding cycle in the clinical setting and has also been found as a sensitive and reliable method in reproducing symptoms of women with OAB and for the detection of DO (16, 17). To the best of our knowledge, urodynamic evaluation of CI with AUM has not been studied so far.

The aim of this study was to investigate the clinical risk factors for CI and its association with urodynamic diagnoses at single voiding cycle AUM performed in the clinical setting.

## Materials and methods

Records of sexually active women with UI at the urogynecology unit of Ankara University Faculty of Medicine, Department of Obstetrics and Gynecology between 2008 and 2022, who completely fulfilled the Turkish validated short form of the Pelvic Organ Prolapse/Urinary Incontinence Sexual Questionnaire (PISQ-12) were reviewed retrospectively. Women were grouped using the 6th question of the PISQ-12; women who answered “never” to this question were considered as continent during coitus and women who reported any urinary leakage at coitus were considered as coital incontinent. Data regarding their routine urogynecology evaluation were retrieved; demographics, patient reported LUTS, clinical findings, incontinence severity, Turkish validated questionnaires for pelvic floor dysfunction symptom bother and quality of life were compared in women with and without CI. As part of the urogynecologic examination in our unit, pelvic floor muscle strength evaluation and simplified POPQ staging were performed as described previously (1, 18). Incontinence severity was evaluated with the Sandvik Incontinence Severity Index. The questionnaires included the short form of the Pelvic Floor Distress Inventory (PFDI-20), Overactive Bladder Awareness Tool (OAB-V8), the PISQ-12, and short form of the Incontinence Impact Questionnaire (IIQ-7).

Records of women who underwent AUM for complicated LUTS were also reviewed; data regarding cystometry findings were retrieved and compared among the groups. In our unit, AUM is performed as the primary urodynamic investigation using the LUNA ambulatory monitoring recorder (MMS™) compatible to the standards of sub-committee of ICS for AUM (19), with a standardized protocol as follows (16).

After excluding urinary tract infections, bowel preparation is performed to ensure good-quality rectal tracing and avoiding artifacts, if required. After spontaneous micturition and mea- surement of PVR by catheterization, a 7F double lumen air-charged single sensor bladder catheter (T-DOC, Laborie™) and a 7F single lumen air-charged rectal catheter (T-DOC, Laborie™) are inserted to measure intravesical and abdominal pressures, respectively. Both catheters are securely taped adjacent to the external meatus of urethra at 12 cm and to anus at 9 cm and are connected to a microcomputer worn over the shoulder, allowing patients to move freely. Each transducer is set to zero atmospheric pressure before each investigation with the patient in standing position. The signal quality of catheters is checked several times with coughing or abdominal straining, before starting recording, with regular intervals during monitoring and before finalizing.

Patients are informed about the use of LUNA event buttons for marking “urinary leakage,” “urgency,” “physical activity,” and “drinking water.” All women are asked drinking 500 ml of water in 30 min at the beginning of AUM. During AUM, all patients are encouraged toward activities or maneuvers that are provocative for their daily urinary symptoms (e.g., listening to running water, hand washing, coughing, sitting, picking up an object from the floor, standing, walking, and jumping) in the special area reserved for AUM in the hospital setting. Urinary leakage during AUM is verified using a pad test.

When the patients are unable to delay voiding, monitoring is ended by a pressure-flow study performed by a PC based wireless uroflowmeter (Flowmaster, MMS™) in a special section of the room to preserve privacy. After the completion of the urodynamic study, all data are transferred from LUNA to the PC; the quality control of traces recorded during both substracted cystometry and pressure-flow study, and interpretation of data are performed by a trained supervisor before the patients leave.

Univariate and multivariate binomial logistic regression analyses were performed to investigate the clinical risk factors including demographics, patient reported symptoms and examination findings associated with CI in the whole study population. As not all women underwent urodynamics, these analyses were performed in the subgroup of women with urodynamic data separately, to evaluate the relationship of urodynamic diagnoses and CI.

### Statistical analysis

Statistical analyses were performed using the SPSS software version 16. The variables were investigated using visual (histograms and probability plots) and analytical methods (Kolmogorov–Smirnov/Shapiro–Wilk test) to determine whether they are normally distributed or not. Continuous variables were presented as mean ± SD and median (range) whereas categorical variables were presented as number and percentage. Descriptive statistics of continuous variables were compared between groups using the Student-t or Mann–Whitney test where appropriate. The Chi-square test or Fisher’s exact test, where appropriate, were used to compare categorical variables between groups. The univariate analyses to identify variables associated with CI was investigated using Chi-square, Fisher exact, Student’s *t*-and Mann–Whitney U tests were appropriate. For the multivariate analysis, the possible factors identified with univariate analyses (all variable with a *p*-value below 0.20) were further entered into the logistic regression analysis to determine independent factors associated with CI. Hosmer–Lemeshow goodness of fit statistics were used to assess model fit. A 5% type-I error level was used to infer statistical significance.

## Results

**FIGURE 1:**
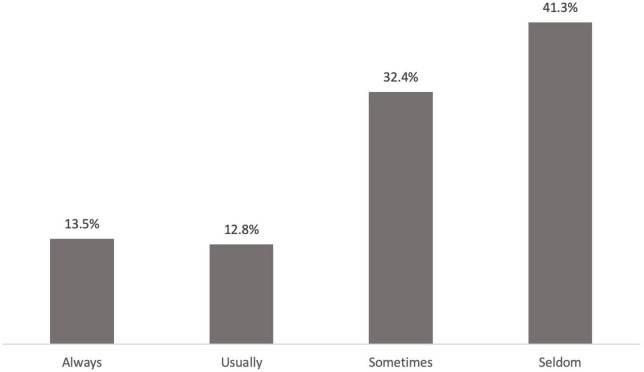
Percentage of women regarding the severity of coital incontinence according to the 6th question of PISQ12 (*n* = 414).

Among all sexually active women with UI who completed the PISQ-12 (*n* = 1,005), 41.2% (*n* = 414) were found to have CI according to the 6th question; 12.44% (*n* = 125) were found to report CI on direct questioning as well, including 9 women (0.896%) volunteering this symptom. Regarding the severity of CI, 13.5% (*n* = 56) of women expressed their symptom as “always,” 12.8% (*n* = 53) as “usually,” 32.4% (*n* = 134) as “sometimes” and 41.3% (*n* = 171) as “seldom” according to the 6th question of PISQ-12 (Figure 1).

Women with CI were significantly younger and menopausal status was lower compared to women with no CI; body mass index (BMI) and cigarette smoking status were also significantly higher in these women (Table 1). Symptoms of SUI, postural UI and insensible loss of urine were found to be significantly higher in women with CI (*p* ≤ 0.004). Women suffering from CI had significantly more daily UI episodes on the 3-day urinary diary (*p* < 0.001), and cough stress test, supine empty stress test (SEST) and Q-tip test positivities were more frequent in women with CI (*p* ≤ 0.001). Advanced stage anterior prolapse (POPQ ≥ Stage 3) was significantly less frequent in these women (*p* = 0.042) (Table 1).

**TABLE 1:** Demographics, patient reported LUTS, urinary diary, physical examination findings in women with and without coital incontinence.

	Coital incontinence (−) (*n* = 591)	Coital incontinence (+) (*n* = 414)	*p*
**Demographics**			
Age (years)			<0.001
Mean ± SD	51 ± 11	49 ± 9	
Median (min–max)	51 (21–88)	49 (24–77)	
Postmenopausal, *n* (%)	320 (54)	183 (44)	0.002
Parity (*n*)			0.470
Mean ± SD	2.7 ± 1.5	2.7 ± 1.4	
Median (min–max)	2 (0–14)	2 (0–10)	
Previous vaginal delivery, *n* (%)	550 (93)	395 (95)	0.122
Body-mass index (kg/m^2^)			0.019
Mean ± SD	29.1 ± 5.0	29.9 ± 4.9	
Median (min–max)	28 (18–48)	30 (20–48)	
Smoking, *n* (%)	84 (14)	79 (19)	0.039
Diabetes, *n* (%)	93 (16)	72 (17)	0.486
Hypertension, *n* (%)	170 (29)	123 (30)	0.746
Previous hysterectomy, *n* (%)	62 (10)	37 (9)	0.416
Previous colporrhaphy, *n* (%)	20 (3)	8 (2)	0.169
Previous anti-incontinence surgery, *n* (%)	27 (5)	22 (5)	0.589
**Patient reported LUTS**			
Nocturia, *n* (%)	393 (66)	282 (68)	0.591
Frequency, *n* (%)	356 (60)	266 (64)	0.197
Urgency, *n* (%)	312 (53)	209 (50)	0.471
Suprapubic pain, *n* (%)	230 (39)	155 (37)	0.635
Stress UI, *n* (%)	304 (51)	262 (63)	<0.001
Urgency UI, *n* (%)	284 (48)	219 (53)	0.131
Postural UI, *n* (%)	59 (10)	92 (22)	<0.001
Insensible loss of urine, *n* (%)	47 (8)	56 (13)	0.004
Nocturnal UI, *n* (%)	51 (9)	49 (12)	0.095
**3-day urinary diary findings**			
Daily fluid intake (L), mean ± SD	2.2 ± 0.9	2.2 ± 0.9	0.854
Daily micturition episodes (*n*), mean ± SD	8.5 ± 3.8	8.4 ± 3.6	0.781
Daily UI episodes (*n*), mean ± SD	2.1 ± 3.2	3.3 ± 3.5	< 0.001
**Physical examination findings**			
POPQ ≥ Stage 3, *n* (%)			
Anterior	110 (19)	57 (14)	0.042
Apical	70 (12)	37 (9)	0.141
Posterior	50 (8)	25 (6)	0.150
Positive Q-tip test, *n* (%)	368 (62)	301 (73)	0.001
Positive cough stress test, *n* (%)	208 (35)	254 (61)	< 0.001
Positive supine empty stress test, *n* (%)	63 (11)	108 (26)	< 0.001
Pelvic floor muscle strength (MOS), median (min–max)	3 (0–5)	2 (0–5)	0.443

UI, urinary incontinence; POPQ, pelvic organ prolapse quantification; MOS, modified oxford score. p < 0.05 statistically significant.

In women with CI, all domains of the questionnaires revealed that UI was more severe, symptom bother was higher and related quality of life was worse (*p* < 0.001). Sexual function in all domains was also impaired in these women (≤0.018) (Table 2).

**TABLE 2:** Scores of the questionnaires in women with and without coital incontinence.

Questionnaires*	Coital incontinence (−) (*n* = 591)	Coital incontinence (+) (*n* = 414)	*p*
Sandvik incontinence severity index	5.9 ± 3.9 4 (1–12)	7.5 ± 3.9 8 (1–12)	<0.001
PFDI-20 total score	97 ± 54 93 (0–230)	126 ± 54 125 (4–284)	<0.001
**UDI-6 scores**			
Total	43 ± 24 42 (0–100)	59 ± 23 60 (0–100)	<0.001
Irritative symptoms	55 ± 33 50 (0–100)	70 ± 29 75 (0–100)	<0.001
Stress symptoms	42 ± 34 37 (0–100)	67 ± 31 75 (0–100)	<0.001
Obstructive symptoms	33 ± 30 25 (0–100)	42 ± 31 37 (0–100)	<0.001
POPDI-6 total score	31 ± 22 29 (0–96)	37 ± 24 33 (0–100)	0.001
CRADI total score	23 ± 19 19 (0–91)	29 ± 20 25 (0–94)	<0.001
**IIQ-7 scores**			
Total	35 ± 29 28 (0–100)	52 ± 30 52 (0–100)	<0.001
Physical activity	40 ± 34 33 (0–100)	58 ± 33 67 (0–100)	<0.001
Travel	33 ± 33 33 (0–100)	50 ± 35 50 (0–100)	<0.001
Social/relationships	32 ± 37 33 (0–100)	49 ± 38 67 (0–100)	<0.001
Emotional health	35 ± 35 33 (0–100)	49 ± 37 50 (0–100)	<0.001
OAB-V8 total score	17 ± 10 16 (0–40)	23 ± 10 23 (0–40)	<0.001
**PISQ-12 scores**			
Total	31 ± 6 31 (7–45)	25 ± 7 25 (2–43)	<0.001
Behavioral/emotive	6 ± 4 6 (0–16)	7 ± 4 6 (0–16)	0.018
Physical	16 ± 3 17 (5–20)	11 ± 4 11 (0–19)	<0.001
Partner-related	8 ± 2 9 (0–12)	7 ± 2 7 (0–11)	<0.001

*Data are presented as Mean ± SD and Median (min–max), p < 0.05 statistically significant. PFDI-20, Short form of the Pelvic Floor Distress Inventory; UDI-6, Short form of the Urogenital Distress Inventory; POPDI, pelvic organ prolapse distress inventory; CRADI, Colorectal anal distress inventory; IIQ-7, Short form of the Incontinence Impact Questionnaire; OAB-V8, Overactive Bladder Awareness Tool; PISQ-12, Short form of the pelvic organ prolapse/urinary incontinence sexual questionnaire.

Among women comprising the whole study population (*n* = 1,005), 552 women were found to have AUM data, including 258 women with CI and 294 women without CI. On comparison of the AUM findings between the groups the duration, maximum cystometric capacities, and the presence of urgency, urgency urinary incontinence (UUI), mixed urinary incontinence (MUI), and DO were similar in both groups (*p* ≥ 0.063), whereas only SUI was significantly higher in women with CI (*p* = 0.018) (Table 3).

**TABLE 3:** Cystometry findings at AUM in women with and without coital incontinence.

Cystometry	Coital incontinence (−) (n = 294)	Coital incontinence (+) (n = 258)	*p*
Duration (minutes)			
Mean ± SD	90 ± 29	93 ± 30	2*0.299
Median (min–max)	82 (35–196)	86 (36–236)	
Maximum cystometric capacity (ml)			
Mean ± SD	456 ± 183	476 ± 200	2*0.252
Median (min–max)	436 (151–1,133)	455 (150–1,114)	
Urgency, *n* (%)	263 (89)	236 (91)	0.422
Urodynamic stress UI, *n* (%)	59 (20)	74 (29)	0.018
Urodynamic urgency UI, *n* (%)	42 (14)	30 (12)	0.355
Urodynamic mixed UI, *n* (%)	95 (32)	103 (40)	0.063
Detrusor overactivity, *n* (%)	160 (54)	136 (53)	0.688

UI, urinary incontinence. p < 0.05 statistically significant.

On univariate analysis, age, menopausal status, BMI, smoking, patient reported SUI, postural UI, insensible loss of urine, anterior POP ≥ stage 3, positive Q-tip test, positive cough stress test and positive SEST were significantly associated with CI (*p* ≤ 0.042) (Table 4). On multivariate analysis, younger age, history of vaginal delivery, smoking, presence of postural UI, positive cough stress test and positive SEST (*p* ≤ 0.041) were found as independent clinical factors associated with CI (Table 4). Urodynamic SUI and MUI (*p* ≤ 0.002) were found as independent urodynamic diagnoses associated with CI (Table 5).

**TABLE 4:** Univariate and multivariate analyses of clinical factors associated with coital incontinence.

	Univariate analysis		Multivariate analysis	
	**Odds ratio (CI)**	***p***	**Odds ratio (CI)**	***p***
**Demographics**				
Age (years)	0.976 (0.963–0.988)	<0.001	0.967 (0.952–0.983)	<0.001
Postmenopausal status	0.671 (0.521–0.864)	0.002		
Previous vaginal delivery	1.550 (0.886–2.711)	0.122	2.127 (1.133–3.992)	0.019
Body-mass index	1.034 (1.005–1.063)	0.019		
Smoking	1.423 (1.016–1.993)	0.039	1.490 (1.016–2.185)	0.041
Previous colporrhaphy	0.563 (0.245–1.290)	0.169		
**Patient reported LUTS**				
Frequency	1.186 (0.915–1.539)	0.197		
Stress UI	1.627 (1.259–2.104)	<0.001		
Urgency UI	1.214 (0.944–1.561)	0.131		
Postural UI	2.576 (1.806–3.674)	<0.001	2.012 (1.345–3.011)	0.001
Insensible loss of urine	1.811 (1.202–2.728)	0.005		
Nocturnal UI	1.421 (0.940–2.150)	0.096		
**Physical examination findings**				
POPQ ≥ Stage 3				
Anterior	0.698 (0.493–0.989)	0.042		
Apical	0.730 (0.480–1.112)	0.141		
Posterior	0.695 (0.423–1.144)	0.150		
Positive Q-tip test	1.614 (1.229–2.120)	0.001		
Positive cough stress test	2.923 (2.254–3.791)	<0.001	2.193 (1.542–3.118)	<0.001
Positive supine empty stress test	2.958 (2.103–4.161)	<0.001	1.756 (1.143–2.699)	0.010

UI, urinary incontinence; POPQ, pelvic organ prolapse quantification. p < 0.05 statistically significant.

**TABLE 5:** Univariate and multivariate analyses of urodynamic diagnoses at AUM associated with coital incontinence.

Urodynamic diagnosis	Univariate analysis		Multivariate analysis	
	**Odds ratio (CI)**	***p***	**Odds ratio (CI)**	***p***
SUI	1.602 (1.082–2.372)	0.018	2.168 (1.399–3.359)	0.001
UUI	0.789 (0.478–1.304)	0.355		
MUI	1.392 (0.982–1.973)	0.063	1.874 (1.268–2.770)	0.002
DO	0.934 (0.668–1.305)	0.688		

SUI, stress urinary incontinence; UUI, urgency urinary incontinence; MUI, mixed urinary incontinence; DO, detrusor overactivity. p < 0.05 statistically significant.

## Discussion

In this study population, the prevalence of CI identified with PISQ-12 was 41.2%, which is in accordance with most previous studies (2, 20–22), confirming the importance of the validated questionnaires in increasing disclosure, and the identification of women with this embarrassing symptom.

Women with CI had higher symptom bother in all domains of the PFDI-20 and the OAB-V8, and more severe urinary incontinence with impaired quality of life and sexual function. These findings are in accordance with the results of the studies of Oh et al. (20) and Pons and Clota (2) who reported worse incontinence symptoms with impaired sexual function and quality of life. Additionally, Gray et al. (21) have reported that women with CI had significant self and partner avoidance of sex, and lower quality of life due to sexual problems using the e-PAQ-PF, in their cohort of 2,312 women attending the urogynaecology clinic.

The relation of patient characteristics with CI have been understudied; few studies have reported clinical risk factors for CI, with contradictory results (4, 5, 8–10, 22). There are studies both reporting no association with age (5, 8, 9, 22) or younger age in women with CI (4, 10). In this study, younger age was found independently related with CI.

It is known that pregnancy and delivery have substantial impact on the pelvic floor, and the impact is greater with vaginal delivery (23). Studies using objective assessments such as ultrasound, MRI, urodynamics, and electrophysiologic tests have already demonstrated that pregnancy and vaginal delivery are associated with descended bladder neck, increased bladder neck mobility, decreased levator ani strength and urethral resistance (24). In this study, vaginal delivery was also found to be a strong independent risk factor related with CI (OR 2,127; 95% CI 1,133–3,992), supporting these findings. Similarly, Illiano et al. (9) have reported that caesarean section was an independent and significant protective factor for CI. On the contrary, Dietz and Subramaniam (8) have found no relation between vaginal delivery and CI.

Smoking was another independent factor associated with CI in the present study, in accordance with the studies of Madhu et al. (10) who reported smoking as a significant risk factor for CI (25). This finding has been attributed to the antiestrogenic effects and lower collagen synthesis related with smoking (10, 26).

In the present study, BMI was found as another associated risk factor for CI, in line with studies which also have shown this relationship (8–10, 22). Indeed, obesity is a known risk factor for UI, and the pathophysiological mechanism has been explained by the negative effects of chronic increased intra-abdominal pressure and oxidative stress from visceral adipose on the collagen content and supportive neuromuscular structures of the pelvic floor (27). However, there are also studies reporting no association (2, 4).

In this study, patient reported symptoms of SUI, postural UI and insensible loss of urine, with positive cough stress test, SEST and Q-tip test were found to be significantly associated with CI, all indicating SUI and possible urethral incompetence, as the predominant mechanism underlying this symptom. Ambulatory urodynamic monitoring of these women during single voiding cycle in the clinical setting also supported that SUI was found as the main urodynamic diagnosis related with CI. Moreover, no relation was found with DO or UUI. Similar results have been reported previously with conventional urodynamics (4, 5, 8). El-Azab et al. (5) additionally showed a significant positive correlation with the severity of SUI and a significant negative correlation with abdominal leak point pressure (ALPP) and emphasized the role of potential urethral incompetence in the etiopathogenesis of CI. In accordance, Dietz and Subramaniam (8) have also found that ALPP and mid-urethral closure pressure (MUCP) were significantly associated factors.

On the other hand, it has previously been reported that CI at penetration is associated with SUI, and that CI during orgasm is associated with DO (6, 7). Contradictorily, Jha et al. (3) have found no association with urodynamic diagnoses, neither at penetration nor orgasm. There are also studies concluding that SUI is the main mechanism associated with all patient reported types of CI (4, 5, 8, 10). None of the studies have performed urodynamics during orgasm except the study of Khan et al. (28) including 3 cases who underwent urodynamic examination prior to and during orgasm. They demonstrated that detrusor contractions may be triggered during orgasm resulting in urethral relaxation and leakage (28); probably because of the loss of the external urethral sphincter reflex and resulting sphincter incompetence. Or, vice versa, leakage due to urethral incompetence may also provoke detrusor contractions, as shown before (29).

The main limitation of the present study is its retrospective design; thus, we were not able to evaluate objective urodynamic measures for urethral incompetence such as ALPP or MUCP. Additionally, we were also not able to evaluate the presence of urethral diverticulum, which may be a related underlying factor for CI. The main strengths are that we investigated the relationship of CI with clinical factors comprehensively in a large cohort of sexually active women with UI, and we evaluated the underlying pathophysiology with ambulatory urodynamics, as a more sensitive tool in the detection of DO.

In brief, women with UI presented very rarely with CI and the use of a validated questionnaire significantly increased the identification of women with this symptom in this study. Women with CI were found to have more severe UI, higher pelvic floor symptom bother, with worse sexual function and related quality of life. Younger age, history of vaginal delivery, smoking, and the presence of postural UI, positive cough stress test and positive SEST were found as independent clinical factors associated with this symptom. Although AUM was the most sensitive urodynamic tool in the detection of DO, no relationship was found. Our results may also suggest that treatment of SUI would also improve their incontinence associated with sexual intercourse. It is also noteworthy to emphasize that 26.3% of the study population were found to have severe CI, whereas the remainder had mild symptoms and the results seem to be more reflective of mild CI. Hence, the underlying mechanism according to severity needs further investigation.

## Conclusion

In conclusion, both clinical and AUM findings supported that CI is a more severe form of UI that it is mainly related with urethral incompetence. Further prospective research is needed to clarify the exact pathophysiologic mechanism.

## Data availability statement

The data analyzed in this study is subject to the following licenses/restrictions: The dataset is not publicly available to preserve individuals’ privacy. Requests to access these datasets should be directed to MS, muratseval@gmail.com.

## Ethics statement

The studies involving human participants were reviewed and approved by the Ankara University School of Medicine, Department of Obstetrics and Gynecology, Institutional Review Boards. Written informed consent was not provided because retrospective design of the study.

## Author contributions

ŞÇ and FD contributed to the design of the study. MS and BV organized the database and performed the statistical analysis. ŞÇ wrote the first draft of the manuscript. All authors contributed to the manuscript revision and approved the submitted version.

## References

[1] Haylen B de Ridder D Freeman R Swift S Berghmans B Lee J An International Urogynecological Association (IUGA)/International Continence Society (ICS) joint report on the terminology for female pelvic floor dysfunction. *Int Urogynecol J.* (2010) 21:5–26. 10.1007/s00192-009-0976-9 19937315

[2] Pons M Clota M. Coital urinary incontinence: impact on quality of life as measured by the King’s Health Questionnaire. *Int Urogynecol J.* (2008) 19:621–5. 10.1007/s00192-007-0490-x 17973067

[3] Jha S Strelley K Radley S. Incontinence during intercourse: myths unravelled. *Int Urogynecol J.* (2012) 23:633–7. 10.1007/s00192-011-1583-0 22237785

[4] Moran P Dwyer P Ziccone S. Urinary leakage during coitus in women. *J Obstet Gynaecol.* (1999) 19:286–8. 10.1080/01443619965084 15512297

[5] El-Azab A Yousef H Seifeldein G. Coital incontinence: relation to detrusor overactivity and stress incontinence. *Neurourol Urodyn.* (2011) 30:520–4. 10.1002/nau.21041 21268103

[6] Hilton P. Urinary incontinence during sexual intercourse: a common, but rarely volunteered, symptom. *Br J Obstet Gynaecol.* (1988) 95:377–81. 10.1111/j.1471-0528.1988.tb06609.x 3382610

[7] Serati M Salvatore S Uccella S Cromi A Khullar V Cardozo L Urinary incontinence at orgasm: relation to detrusor overactivity and treatment efficacy. *Eur Urol.* (2008) 54:911–5. 10.1016/j.eururo.2007.11.008 18036728

[8] Dietz H Subramaniam N. Is coital incontinence a manifestation of urodynamic stress incontinence or detrusor overactivity? *IUJ.* (2022) 33:1175–8. 10.1007/s00192-021-04809-8 33938964

[9] Illiano E Mahfouz W Giannitsas K Kocjancic E Vittorio B Athanasopoulos A Coital incontinence in women with urinary incontinence: an international study. *J Sex Med.* (2018) 15:1456–62. 10.1016/j.jsxm.2018.08.009 30245022

[10] Madhu M Hashim H Enki D Yassin M Drake M. Coital incontinence: what can we learn from urodynamic assessment? *Urology.* (2015) 85:1034–8. 10.1016/j.urology.2015.02.007 25917729

[11] Radley S Rosario D Chapple C Farkas A. Conventional and ambulatory urodynamic findings in women with symptoms suggestive of bladder overactivity. *J Urol.* (2001) 166:2253–8. 11696746

[12] Dokmeci F Seval M Gok H. Comparison of ambulatory versus conventional urodynamics in females with urinary incontinence. *Neurourol Urodyn.* (2010) 29:518–21. 10.1002/nau.20821 19731314

[13] van Koeveringe G Rahnama’i M Berghmans B. The additional value of ambulatory urodynamic measurements compared with conventional urodynamic measurements. *BJU Int.* (2010) 105:508–13. 10.1111/j.1464-410X.2009.08790.x 19673868

[14] Rademakers K Drossaerts J Rahnama’i M van Koeveringe G. Differentiation of lower urinary tract dysfunctions: the role of ambulatory urodynamic monitoring. *Int J Urol.* (2015) 22:503–7. 10.1111/iju.12723 25711671

[15] Cantu H Sharaf A Bevan W Hassine A Hashim H. Ambulatory urodynamics in clinical practice: a single centre experience. *Neurourol Urodyn.* (2019) 38:2077–82. 10.1002/nau.24153 31471918

[16] Dokmeci F Cetinkaya S Seval M Dai O. Ambulatory urodynamic monitoring of women with overactive bladder syndrome during single voiding cycle. *Eur J Obstet Gynecol Reprod Biol.* (2017) 212:126–31. 10.1016/j.ejogrb.2017.03.023 28355584

[17] Seval M Çetinkaya Ş Kalafat E Dökmeci F. A prediction model for detrusor overactivity at ambulatory urodynamics in women with urinary incontinence. *Eur J Obstet Gynecol Reprod Biol.* (2020) 251:156–61. 10.1016/j.ejogrb.2020.05.035 32505788

[18] Laycock J. Clinical evaluation of the pelvic floor. In: Schussler B Laycock J Norton P Stanton S editors. *Pelvic Floor re-Education, Principles and Practice.* London: Springer-Verlag (1994). p. 39–51.

[19] van Waalwijk van Doorn E Anders K Khullar V Kulseng-Hanssen S Pesce F Robertson A Standardisation of ambulatory urodynamic monitoring: report of the standardisation sub-Committee of the international continence society for ambulatory urodynamic studies. *Neurourol Urodyn.* (2000) 19:113–25. 10679828 10.1002/(sici)1520-6777(2000)19:2<113::aid-nau2>3.0.co;2-#

[20] Oh S Choo M Kim H Kim J Lee J Yun J Generic and disease-specific health-related quality of life in women with coital incontinence: a prospective, multicenter study. *Gynecol Obstet Investig.* (2007) 65:62–7. 10.1159/000107978 17851252

[21] Gray T Li W Campbell P Jha S Radley S. Evaluation of coital incontinence by electronic questionnaire: prevalence, associations and outcomes in women attending a urogynaecology clinic. *Int Urogynecol J.* (2018) 29:969–78. 10.1007/s00192-017-3380-x 28620792

[22] Lau H Huang W Su T. Urinary leakage during sexual intercourse among women with incontinence: incidence and risk factors. *PLoS One.* (2017) 12:e0177075. 10.1371/journal.pone.0177075 28542221 PMC5443475

[23] Blomquist J Muñoz A Carroll M Handa V. Association of delivery mode with pelvic floor disorders after childbirth. *JAMA.* (2018) 320:2438–47. 10.1001/jama.2018.18315 30561480 PMC6583632

[24] Van Geelen H Ostergard D Sand P. A review. *Int Urogynecol J.* (2018) 29:327–38. 10.1007/s00192-017-3540-z 29332252

[25] Madhu C Enki D Drake M Hashim H. The functional effects of cigarette smoking in women on the lower urinary tract. *Urol Int.* (2015) 95:478–82. 10.1159/000438928 26452108

[26] Bump R McClish D. Cigarette smoking and urinary incontinence in women. *Am J Obstet Gynecol.* (1992) 167:1213–8. 10.1016/s0002-9378(11)91691-3 1442969

[27] Doumouchtsis S Loganathan J Pergialiotis V. The role of obesity on urinary incontinence and anal incontinence in women: a review. *BJOG.* (2022) 129:162–70. 10.1111/1471-0528.16848 34312978

[28] Khan Z Bhola A Starer P. Urinary incontinence during orgasm. *Urology.* (1988) 31:279–82. 10.1016/0090-4295(88)90160-4 3347980

[29] Jung S Fraser M Ozawa H Yokoyama O Yoshiyama M De Groat W Urethral afferent nerve activity affects the micturition reflex; implication for the relationship between stress incontinence and detrusor instability. *J Urol.* (1999) 162:204–12. 10.1097/00005392-199907000-00069 10379788

